# P-1143. Etiology and resistance phenotypes of blood-culture proven neonatal sepsis from two large NICUs in Mexico: a multicenter three-year retrospective analysis

**DOI:** 10.1093/ofid/ofae631.1330

**Published:** 2025-01-29

**Authors:** Abraham P Garza-Castro, Ana Ballesteros-Suarez, Lindsay A Concha-Mora, Ericka Paulina Prieto-Baca, Oscar Tamez-Rivera

**Affiliations:** Residencia de Pediatría, Programa Multicéntrico de Especialidades Médicas. ITESM-SSNL. Tecnológico de Monterrey, Escuela de Medicina y Ciencias de Salud., Santiago, Nuevo Leon, Mexico; Departamento de Medicina, Tecnológico de Monterrey, Escuela de Medicina y Ciencias de la Salud, Monterrey, México, Monterrey, Nuevo Leon, Mexico; Pediatric Residency Program, Programa Multicéntrico de Especialidades Médicas ITESM- SSNL, Tecnológico de Monterrey. Escuela de Medicina y Ciencias de la Salud. Monterrey, México, Monterrey, Nuevo Leon, Mexico; Departamento de Medicina, Tecnológico de Monterrey, Escuela de Medicina y Ciencias de la Salud, Monterrey, México, Monterrey, Nuevo Leon, Mexico; Tecnologico de Monterrey, Escuela de Medicina y Ciencias de la Salud, Monterrey, Nuevo Leon, Mexico

## Abstract

**Background:**

Neonatal sepsis is a significant cause of morbidity and mortality in newborns. Etiology depends on multiple factors, particularly regional epidemiology. Current international guidelines are based on data from developed countries, which may not reflect the situation of developing regions. Microbiological surveillance in Mexican NICUs is limited, which may lead to inappropriate antimicrobial use.

**Figure:**
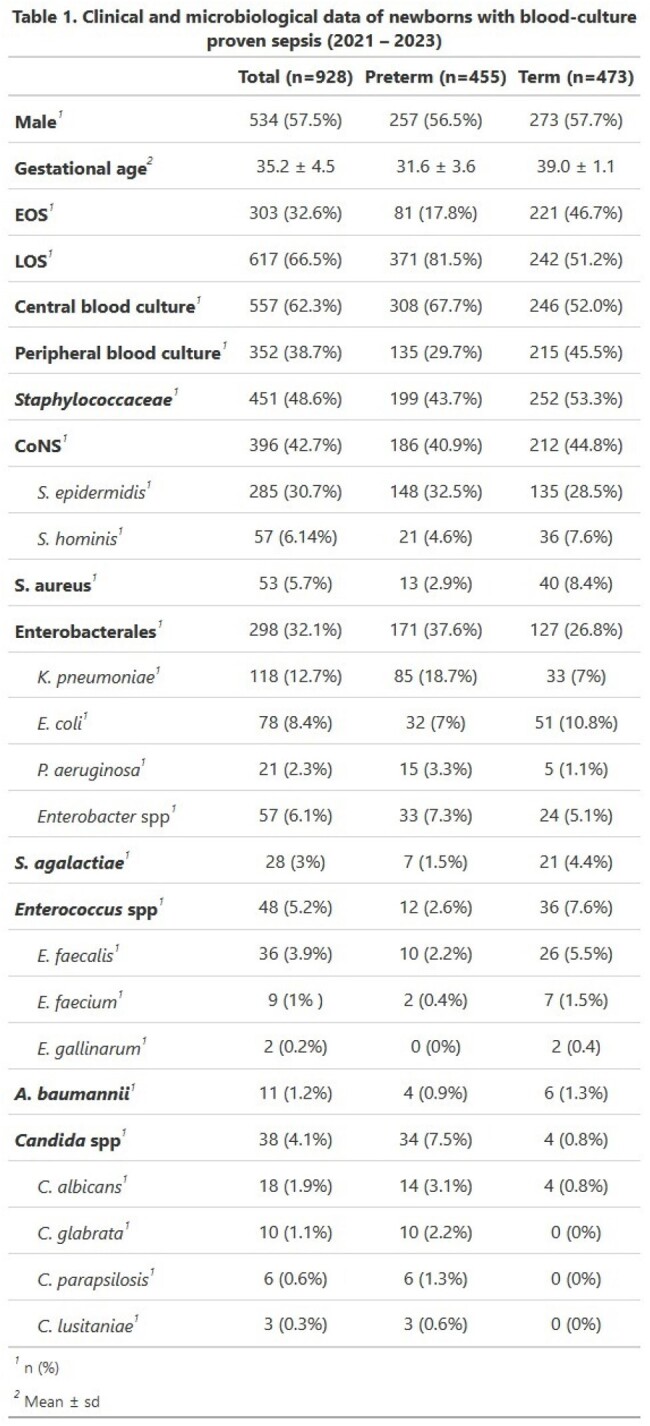

**Methods:**

This is a retrospective multicenter study conducted in two NICUs from NL, Mexico (2021-2023). Clinical and microbiological data on ≤28-day newborns with blood culture-proven neonatal sepsis were collected. Neonatal sepsis was classified as early (≤3 days) and late onset ( >3 days). Descriptive statistical analyses were performed to assess microbiologic etiologies, as well as clinically-relevant resistance phenotypes (VITEK® 2).

**Figure:**
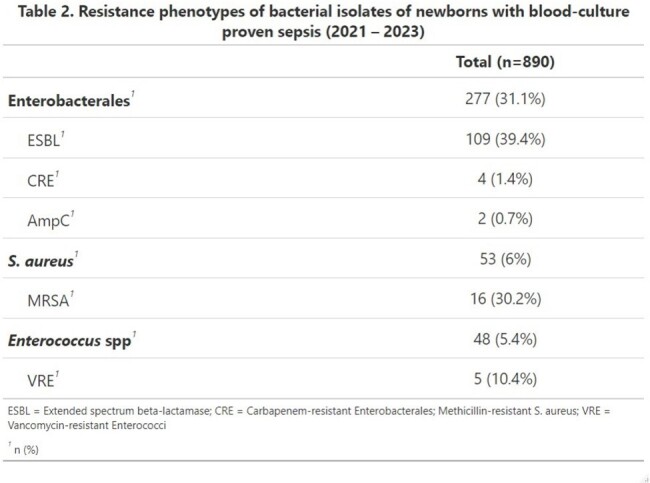

**Results:**

A total of 928 blood cultures from 775 patients were included. 49.0% were preterm. Mean gestational age was 35.2. Most episodes (66.9%) were classified as late-onset sepsis (Table 1). Staphylococcaceae were the most commonly identified family (48.6%). Coagulase-negative Staphylococci were the most common group (42.7%), being *S. epidermidis* the most common. Gram-negative bacilli were the second most common cause of neonatal sepsis (33.7 %), mostly due to *K. pneumoniae* (12.7%). Low prevalence of GBS was observed (3.0%). Fungal (4.1%) and enterococcal (5.2%) isolations were uncommon. The prevalence of ESBL-producing Enterobacterales were 39.4%; MRSA was 30.2% (Table 2). 4 cases (1.4%) of carbapenem-resistant Enterobacterales (CRE) and 2 cases (0.7%) of AmpC Enterobacterales were identified. Notably, 5 cases (10.4%) of vancomycin-resistant Enterococci (VRE) were identified.

**Conclusion:**

The study reported clinical and microbiological data of blood culture-proven neonatal sepsis. In contrast with U.S. data, this study showed a lower prevalence of GBS and a significant presence of Gram-negative pathogens, alongside notable resistance profiles such as ESBL-producing Enterobacterales and VRE. Antimicrobial surveillance in developing regions is necessary in order to recognize the most common etiologies and develop treatment guidelines according to local epidemiology. VRE is a worldwide concern and should be closely monitored.

**Disclosures:**

**All Authors**: No reported disclosures

